# The effects of theta-burst stimulation on sleep and vigilance in humans

**DOI:** 10.3389/fnhum.2014.00420

**Published:** 2014-06-12

**Authors:** Armand Mensen, Corina Gorban, Marcel Niklaus, Eva Kuske, Ramin Khatami

**Affiliations:** ^1^Department of Sleep Medicine, Clinic BarmelweidBarmelweid, Switzerland; ^2^Department of Medicine, University of ZurichZurich, Switzerland

**Keywords:** transcranial magnetic stimulation, theta-burst stimulation, vigilance, sleep, pre-frontal cortex, associative visual cortex

## Abstract

Repetitive transcranial magnetic stimulation (TMS) has become a popular tool to modulate neuronal networks and associated brain functions in both clinical and basic research. Yet few studies have examined the potential effects of cortical stimulation on general levels of vigilance. In this exploratory study, we used theta-burst protocols, both continuous (cTBS) and intermittent (iTBS) patterns, to examine whether inhibition or excitation of the left dorso-lateral prefrontal cortex (dlPFC) was able to induce reliable and acute changes to vigilance measures, compared to the left dorso-lateral associative visual cortex (dlAVC) as a control site in line with previous work. Partially sleep restricted participants underwent four separate sessions in a single day, in a between subjects design for TBS stimulation type and within subjects for locaton, each consisting of maintenance of wakefulness test (MWT), a sleep latency test, and a psychomotor vigilance task (PVT). TBS significantly affected measures of sleep consolidation, namely latency to sleep stage 2 and sleep efficiency, but had no effects on sleep drive or psychomotor vigilance levels for either TBS type or location. Contrary to our initial hypothesis of the dlAVC as a control site, stimulation to this region resulted in the largest differential effects between stimulation types. Moreover, the effect of TBS was found to be consistent throughout the day. These data may provide the basis for further investigation into therapeutic applications of TBS in sleep disorders.

## Introduction

Transcranial magnetic stimulation (TMS) has become a well-established tool in research and is increasingly being applied in clinical situations. Repetitive TMS (rTMS) has been shown as a promising treatment option of various neurologic and psychiatric disorders. Recent evidence from rTMS studies lends support to the idea that cortical stimulation may be able to affect vigilance states and levels by modifying the release of neurotransmitters. For instance, the availability of dopamine which is implicated in the maintenance of sleep and wake (Boutrel and Koob, 2004), can be influenced using TMS. Strafella et al. have investigated how certain patterns of magnetic stimulation over the cortex can have pronounced effects on sub-cortical dopamine levels (Strafella et al., 2001, 2003). In their initial study they found a significant release of dopamine in striatal regions after a rTMS protocol to the left dorso-lateral prefrontal cortex (dlPFC), but no specific release after stimulation of the control site, the left dorso-lateral associative visual cortex (dlAVC). More recently theta-burst TMS (TBS), a pattern of stimulation shown to be effective at inducing longer lasting changes to cortical levels of excitation (Huang et al., 2005), and in particular intermittent TBS (iTBS) of the left dlPFC has been shown to significantly reduce levels of striatal dopamine, presumably by decreasing cortical excitability (Ko et al., 2008).

Research on the clinical benefits of TMS for patients with medication-resistant, major depressive disorder (George et al., 2013), as well as Parkinson’s Disease (Elahi et al., 2009; Benninger et al., 2011) may also provide links to the vigilance system. These studies aimed at improving affective symptoms and motor functioning but both these diseases exert profound effects on sleep and wakefulness with insomnia and fatigue common in depression while excessive sleepiness is associated with Parkinson’s (Riemann et al., 2001; Comella, 2007). Treatment of these disorders using TMS may rely on its effect on sub-cortical neurotransmitters described above. On the other hand, sleep deprivation has also been shown to have a positive acute effect on depression (Hemmeter et al., 2010), and also increases the amount of available dopamine in the striatum and thalamus (Volkow et al., 2008). Therefore, it may also be hypothesized that the clinical effect of TMS is mediated by an acute effect on sleep and wakefulness.

The term *vigilance* is largely used as an umbrella description of an individual’s ability to sustain attention as well as both their own perception and the more objective measures of sleepiness. At a clinical evaluation, the individual aspects of vigilance are often assessed using a standardized battery of tests. The maintenance of wakefulness test (MWT) consists of sitting in a darkened room for up to 40 min while maintaining a waking state with minimal external stimulation (Sullivan and Kushida, 2008). Participants who cannot remain awake for at least 20 min are generally considered to have a decreased ability to sustain wakefulness. In a similar setting, the propensity for sleep can be measured but allowing the participant to lie down and sleep if possible. From this test, the amount of time it takes the participant to initiate sleep, measured as the latency to stage 1 non-rem sleep (N1), as well as the latencies to deeper stages of sleep and rapid eye movement (REM) sleep can be assessed (Littner et al., 2005; Coelho et al., 2011). As this test is usually done several times during the day is referred to as the multiple sleep latency test (MSLT). Latencies of lower than 5 min to N1 are considered a pathological sign of increased ability to fall asleep. Additionally sleep drive may be measured by looking at the power in the lower frequency bands of electroencephalography (EEG) measures over the course of prolonged nap or night time sleep, or even examining certain characteristics of the individual slow waves themselves (Riedner et al., 2007). Lastly, the psychomotor vigilance task (PVT) is also often used in the standard battery and consists of a game-like task for sustained attention whereby the participant must fixate on a displayed timer and press a button as soon as the timer starts to count (Dinges et al., 1997; Drummond et al., 2005). Decreased levels of vigilance will not only lead to slower mean reaction times, but more specifically affect the slowest 10% of measurements while the fastest 10% of times often remains unchanged which is thought to reflect the momentary changes to levels of attention over the course of the test.

Three studies to date have made strides to directly address the question of whether cortical stimulation may have effects on vigilance levels. Over a decade ago, Cohrs et al., under the hypothesis that the effect of TMS in depression was mediated by effects on REM sleep, examined healthy participants using a high frequency repetitive pattern to induce excitatory changes of the underlying brain areas (Cohrs et al., 1998). Polysomnography of the night sleep following evening stimulation showed a significant delay of REM sleep latency when rTMS was compared to sham stimulation; an effect which was particularly pronounced after rTMS of the dlPFC. Shortly after this study, Graf et al. sought to reproduce these results as well as examine potential changes in the frequency power spectrum of the EEG recordings during sleep (Graf et al., 2001). Although they were unable to find any reduction in REM latency, they did report longer overall latencies to sleep onset and a lower percentage of slow-wave-sleep in nights after stimulation to the dlPFC compared to sham stimulation. Moreover, the study found no reliable changes to frequency power following rTMS. Recently, Rosenquist examined long-term changes in sleep activity in a large cohort of depressed patients using real and sham rTMS of the dlPFC and a variety of sleep questionnaires. Six weeks after TMS treatment, overall patients did not show any effect on subjective sleep measures. However, in a subgroup of patients that showed reliable improvement to their depression symptoms did also show a concurrent improvement to sleep parameters, although the authors argue that this is a reflection on the efficacy of treatment, and not on the influence of TMS on sleep directly (Rosenquist et al., 2013).

A few major limitations surround each of these studies. Firstly, the long-term effects of traditional rTMS methods are usually limited to no longer than 20–30 min (Maeda et al., 2000); yet its influence on sleep wasn’t measured until well after this active period (e.g., earliest measurement was 80 min prior to lights off) (Graf et al., 2001). Moreover, the rTMS protocols used tend to produce highly variable inter-individual results, especially without a guided neuronavigation system which could more accurately locate the dlPFC (Ahdab et al., 2010). Finally, sleep parameters themselves also tend to be highly variable, even for the same individual across different nights. It is therefore plausible that in previous studies the effect of TMS was masked by the individual variability of the measures. Furthermore, these studies have exclusively used TMS protocols which are thought to induce a hyper-excitation of the cortex and sham stimulation as a control. Hence, the effects of cortical inhibition remain elusive, as well as whether other cortical areas have the potential to affect vigilance.

Here we sought to explore the possibility that cortical stimulation using the more efficient TBS protocols (Huang et al., 2005) could reliably affect a wide variety of vigilance measures in the highly acute stage post-stimulation. Given that potential effects may be attributed to sub-cortical dopamine release, we hypothesized that an excitation of the dlPFC would lead to an increase in vigilance measures while inhibition of the underlying cortex would lead to a decrease in vigilance. Stimulation of the dlAVC, used a control site, was thus not expected to show any effects as no dopamine is involved (Strafella et al., 2001).

## Materials and methods

### Participants

Twenty-four participants took part in this single-day study at the sleep center in the Clinic Barmelweid, Switzerland. Participants were screened for possible sleep disorders using a validated sleep questionnaire enquiring about sleep-wake habits, daily fatigue, symptoms suggestive of sleep breathing disorders, narcolepsy, various parasomnias, insomnia, and disturbances of the sleep-wake rhythm. Participants aged ranged from 18 to 45 with a mean age of 26.4 years (SD 7.67), and were all right-handed by self-report. Participants had a mean body mass index of 24.1 (SD 4.36), and a mean Epworth Sleepiness Score (ESS), of 8.7/24 (SD 3.05). All participants were asked to try and not sleep for more than 3 h the night before the experimental day, and not to consume any caffeine or energy-related drinks up to 24 h prior to the experiment. The precise length of sleep restriction was not explicitly controlled for. This acute sleep restriction was made in order to maximize the chances that the participants would fall asleep at some point during the experimental session and reduce ceiling effects. Ethical approval was obtained from the Cantonal Ethical Committee of the Canton Aargau in Switzerland, and all participants signed a written consent form.

### TMS

TMS was applied using the MagPro X100 by MagVenture and a water-cooled, figure-of-eight coil. Cortical sites were found and reproduced using MRI-guided neuronavigation (Advanced Neuro Technology, Enschede, Netherlands) with individual specific MRI images when possible (7/24 subjects), or by a head shape adjusted standardized image (Ahdab et al., 2010). Although an individual MRI for each participant is clearly ideal, the use of the individually adjusted standardized model provides substantial improvements in the consistency of the stimulation location throughout the TBS session, as well as ensuring the precise repetition of location across naps. Due to the common muscle involvement when using TMS over these areas, stimulation intensity was slowly increased from 10% in 5% intervals until 45% or the participant indicated that stimulation level was disturbing. The average intensity values (mean = 37.3%, *SD* = 1.87), corresponded to approximately 80–90% of the participant’s resting motor-evoked potential when measured (6/24 participants). This method was employed so as to minimize discomfort during the stimulation which would likely affect the participant’s ability to fall asleep directly after stimulation. Previous work has demonstrated that consistent cortical responses can be obtained at intensities much lower than that needed to induce a motor response, although we recognize that different cortical areas are likely to have different thresholds of activation (Kähkönen et al., 2005).

TBS consisted of a train of 3 TMS pulses separated by 20 ms (50 Hz), where each train of 3 pulses is separated by 200 ms. iTBS consists of 2 s of TBS followed by 8 s of no stimulation for a total of 600 pulses, a protocol which has been shown to produce a long lasting excitation of the cortex; while cTBS consists of a total of 600 pulses, but without the 8 s pause, which has been shown to be an effective inhibitor of cortical activity (Huang et al., 2005; Ko et al., 2008). No adverse effects (e.g., headache, dizziness), were reported by any participant after any of the TBS protocols.

### Experimental design

**Figure 1:**
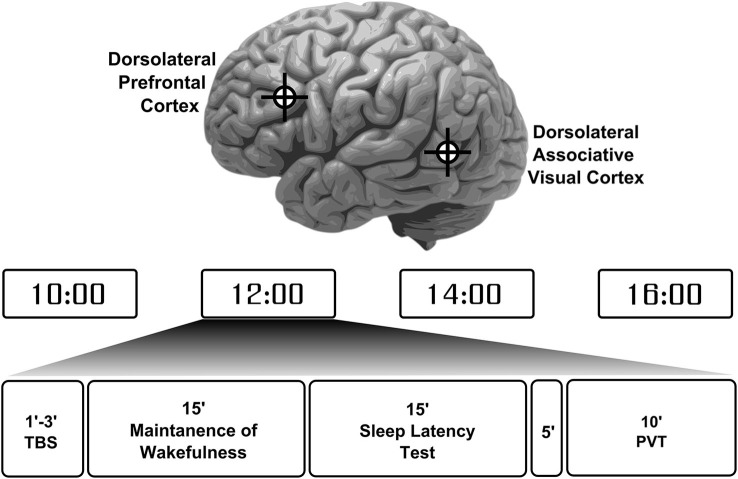
**Participant’s experiment protocol and session overview**. Participants completed a total of 4 sessions throughout the day in evenly spaced times between 10 am to 4 pm. Each session started with theta-burst stimulation (TBS) to either the left dorsolateral prefrontal cortex or the left dorsolateral associative visual cortex in an alternating fashion with the location for first stimulation chosen at random. These locations are depicted on the image of the brain at the top. Each participant received either a continuous or intermittent TBS protocol. Following stimulation participants were required to try to maintain wakefulness for 15 min and then were allowed to sleep for another 15 min. After a 5 min break a psychomotor vigilance task (PVT) was performed to measure participants’ basic reaction times and vigilance levels.

The 24 participants were randomly assigned to one of two stimulation type groups. One group (10 participants) received iTBS while the other group (14 participants), underwent cTBS. Participants performed 4 separate sessions during the day of the experiment starting from 10 am to 4 pm at a 2-h interval (Figure 1). In two of the four experimental sessions (either the first and the third, or the second and fourth), the coil was positioned over the dlPFC (talairach coordinates: *x* = −40, *y* = 32, *z* = 30), tangentially to the scalp with the handle pointing backwards, thus inducing a current flow from posterior to anterior. For the other two sessions, the coil was placed over the left lateral associative visual cortex (dlAVC, talairach coordinates: *x* = −56, *y* = −58, *z* = −3), with the handle pointing forwards, corresponding to the intersection of Brodmann areas 37 and 39. These locations were chosen based on the talaraich coordinates used in Strafella et al. (2001). The dlAVC is considered a control target here and was preferable to using sham or vertex stimulation because in this way, a separate cortical area undergoes similar local neuronal changes induced by the TBS as opposed to a complete lack of neural changes after sham or vertex stimulation.

### Measuring sleep and vigilance

A custom sleep-test battery was constructed consisting of two sleep-related tests followed by a PVT to measure the participant’s level of alertness during wake (Drummond et al., 2005). Initially, a 15 min MWT was conducted where the participant was asked to remain awake while sitting on a bed in a dark, sound-proof room. Immediately after, a second 15 min MSLT was conducted where the participants were able to lie down in bed and were told that they should close their eyes and try not to resist sleep. The combination of these three tests allows for a more objective measurement of the participant’s overall vigilance level and state. All throughout the session, EEG was recorded from three sites; F4, C4 and O2; which were referenced against the contralateral mastoid (A1). The ground electrode was placed on the ipsilateral mastoid A2. Eye movements and blinks were also recorded from a single electrooculogram (EOG) electrode placed just below the right eye and also referenced against A1. Electrode impedances were kept below 10 kΩ. This minimal setup was chosen as to place the least amount of burden on the participant while still being sufficient to effectively score sleep stages in combination with the video recording.

Sleep data was scored by two independent, experienced scorers who were kept blind to the preceding TBS type, in 30 s epochs using the criteria defined by the AASM (Iber et al., 2007). For the MWT, participant’s latency to the first episode of micro-sleep was recorded. This was defined as: at least 5 s of diminished occipital alpha activity unrelated to eyes opening; increase in theta activity in either C4 or F4; and/or slow wave eye movements, as well as the latency to a full stage of N1 sleep (since most participants did not reach a complete stage of N1, only the microsleep latency was further analyzed). For the subsequent MSLT test, participant’s sleep latency to the first N1 and N2 stage was recorded, as well as their total sleep efficiency, measured as the total sleep time (including N1) in relation to their total time in bed. For participants who did not sleep during the tests, the maximum 15 min was used as the sleep latency in the analyses. Although setting these values to the maximal allotted time to sleep may underestimate any wake-promoting effects of TBS; it is nonetheless preferable to designating the session as missing as this would both overestimate potential sleep-promoting effects while unnecessarily reducing statistical power. Moreover, sleep latencies of more than 15 min are considered to be in the clinically normal range.

Once the 30 min were complete, participants were required to sit-up in bed and asked about their level of sleepiness on a 7-point scale and whether they thought they had slept (the Stanford Sleepiness Scale; SSS). If they believed they slept, the participant was asked how long it took them and for how many minutes they slept in total. These values were taken as additional subjective measures of sleep. Five minutes following awakening the participants completed the PVT, from which mean reaction time as well as the fastest and slowest 10% of trials were taken as a measure of vigilance as these measures may be more telling than the overall reaction time.

### Statistical analysis

Given the distinct units that each of the dependent variables has, making direct analysis impossible, each variable was transformed into a *z*-score. The population mean and standard deviation were estimated from all the naps, independent of participant or TBS intervention. The population means was then subtracted from the score for each nap, and divided by the standard deviation to give the transformed *z*-score for each nap. Multiple comparisons and inflated false-positive errors are of principle concern when analyzing the 10 distinct measures of sleep and vigilance. In order to minimize these issues, the 10 measures were aggregated into 4 categories: representing the theoretical relationships among the variables. “Sleep drive” consisted of aggregate scores of latency to micro-sleep in the MWT and MSLT as well N1 latency. “Sleep consolidation” was constructed by latency to N2 and the sleep efficiency, such that higher scores indicated a higher sleep consolidation. “Psychomotor vigilance” was the mean of *z*-scores from the mean, and top and bottom 10% of PVT data. Finally, “subjective influence” combined the inverse SSS, with the perceived latency and inverse of perceived duration of sleep such that higher scores reflected increased feeling of sleepiness.

A linear mixed model approach was used to analyze each measured parameter independently using SPSS (Version 17.0., Chicago: SPSS Inc.). This test type was preferable over the more common analysis of variance approach because: we could account for any missing variable from single sessions in the analysis without completely eliminating the participant altogether (although only a total of 3 individual sessions were missing sleep data for different participants); the covariance structure between the sessions themselves could be explicitly specified and accounted for (autoregressive order 1); and the location of stimulation for each repeated nap could be explicitly stated. Nap (session 1 through 4), location (dlPFC, or dlAVC), and TBS-type (continuous or intermittent) were included as main effects, as well as the two-way interaction between TBS-type and stimulation location and finally the three-way interaction between nap, TBS-type, and location. As baseline levels of these parameters were bound to vary from person to person, the intercept of each participant was further included as a random effect.

Our initial analysis was a multivariate approach which treated the 4 categorical measures as a multivariate case of mixed modeling. By analyzing all dependent variables under a single analytical model we ensure that the false positive rate remains equal to the significance values given by this single test; thus eliminating the multiple comparisons issue at the highest level. At the next stage, each categorical measure was subjected to the identical mixed model approach but as multiple single dependent measures in order to determine the underlying cause of any potential multivariate significant factors. Finally, each of the 10 dependent measures was analyzed on its own as a separate test to examine whether a single measure significantly accounted for potential categorical significant results.

## Results

### Effects on the categorical variables

The highest order multivariate mixed model approach examining the four categorical dependent variables (sleep drive, sleep consolidation, psychomotor vigilance, subjective influence), showed a significant interaction for the factors TBS type and Location of stimulation (*F*_4,214.4_ = 5.257, *p* < 0.001). That is, whether TBS Type, continuous or intermittent had an effect on the measure, depended on the location of stimulation (dlPFC or dlAVC). The analysis also indicated a significant independent effect of nap time (*F*_12,240.1_ = 1.834, *p* = 0.044), such that certain measures showed a significant effect of the time of day the test took place, but which did not have an influence over the TBS type or location of stimulation.

**Figure 2:**
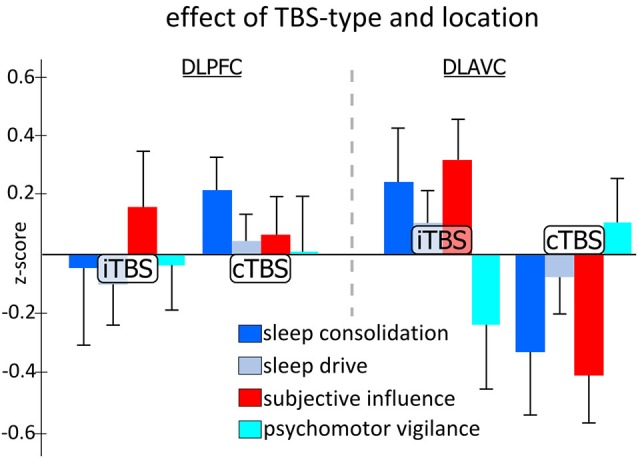
**Effect of theta-burst stimulation on aggregated categorical measures of sleep and vigilance across the two stimulation locations**. Each measure represents the aggregated *z*-scores from various measurements taken during the experiment independent of the time of day the testing occurred. Statistical analysis indicated specific significant differences between TBS-type, either continuous TBS (cTBS) or intermittent TBS (iTBS), for stimulation of the left dorsolateral associative visual cortex (dlAVC), and also for cTBS stimulation between the dorsolateral prefrontal cortex (dlPFC) and dlAVC, for both measures of sleep consolidation and subjective influence. Error bars indicate the standard error for each measure at each location and TBS-type independently.

Figure 2 shows the pattern of effects for the TBS-type and location interaction for each of the categorical variables while Table 1 presents an overview of the results of the mixed model analysis for each categorical measure. Examining each categorical measure independently revealed that the significant Type by Location interaction found in the multivariate test above was most likely caused by both the significant interaction for the “sleep consolidation” aggregate measure (*F*_1,39.5_ = 6.940, *p* = 0.012) and the “subjective influence” measure (*F*_1,49.8_ = 7.001, *p* = 0.011). *Post-hoc* comparisons for the interaction for sleep consolidation using the multivariate analysis and corrected for multiple comparisons using the Sidak procedure, showed that the interaction was driven by both a location difference in the group receiving cTBS (*F*_1,223_ = 10.871, *p* = 0.001), and also TBS type differences for dlAVC stimulation (*F*_1,172_ = 5.389, *p* = 0.021), such that cTBS to the dlAVC produced the least consolidated sleep opposed to any other variant. As with sleep consolidation, subjective influence showed both a location effect of cTBS (*F*_1,223_ = 7.964, *p* = 0.005) and a TBS type difference for the dlAVC location (*F*_1,172_ = 8.189, *p* = 0.005).

**Table 1:** **Statistical overview: effects of stimulation on categorical measures**.

**Category**	**Nap**	**TBS-Type**	**Type by location**	**Nap by type by location**
Sleep Drive	*F* = 0.10	*F* = 0.019	*F* = 2.129	*F* = 0.749
	*p* = 0.921	*p* = 0.468	*p* = 0.154	*p* = 0.663
Sleep Consolidation	*F* = 2.635	*F* = 0.474	*F* = 6.940	*F* = 1.153
	*p* = 0.059*^t^*	*p* = 0.498	*p* = 0.012*	*p* = 0.342
Vigilance	*F* = 0.080	*F* = 0.408	*F* = 2.900	*F* = 1.473
	*p* = 0.970	*p* = 0.529	*p* = 0.093*^t^*	*p* = 0.183
Subjective Influence	*F* = 2.654	*F* = 3.672	*F* = 7.001	*F* = 0.775
	*p* = 0.057*^t^*	*p* = 0.067*^t^*	*p* = 0.011*	*p* = 0.639

The first column indicates the dependent variables analyzed using a mixed model approach, reflecting aggregated z-scores of multiple individual parameters measured during the testing. Sleep drive reflects the latencies to microsleep episodes in the maintenance of wakefulness tests, as well as the latency to stage N1 sleep in the multiple sleep latency tests. Sleep consolidation is the mean of the z-scores for stage N2 latency and the inverse of the total sleep time during the nap. Vigilance is the aggregated scores for the individual measures described by the psychomotor vigilance task while subjective influence combined the scores for the perceived sleepiness of the participant along with their estimated latencies and sleep duration for each nap. The last four columns indicate the F-values and subsequent significance levels of the main factors of nap and TBS type, as well as the two-way interaction between type of stimulation and the location of stimulation, and lastly the three-way interaction between all factors in the model. t indicates a trend level significance while * is indicative of a statistically significant result.

Although no other measure showed significant effects for any other factor or interaction of factors, the univariate mixed model analysis examining “subjective influence” found an additional trends worth noting. For the TBS type factor (*F*_1,24.57_ = 3.672, *p* = 0.067), *post-hoc* analysis revealed that the *z*-scores for iTBS were generally higher than for cTBS, indicating participants subjectively felt less awake following the iTBS protocol which may simply reflect the methodological difference that iTBS takes approximately 3 min to complete as opposed to just 48 s for cTBS.

Although only the sleep consolidation and subjective influence measures found significant effects found for the interaction of TBS type and stimulation location, it does not directly entail that the effect was specific to these measures since other measures may demonstrate similar patterns of effects but nevertheless not reach significance due to increased variability or reduced strengths. In order to test the specificity of the results to the measures of sleep consolidation and subjective influence, we computed the mean difference between stimulation locations across all four naps (given there was no three-way interaction found), for each participant and ran the multiple mixed model approach with planned comparison tests between the categorical measures variables split by TBS-type. As expected from the full mixed model, there was a significant effect of TBS-type (*F*_4,42.3_ = 5.389, *p* = 0.001), while the uni-variate *post-hoc* comparison tests indicated this was driven by differences in the categorical measures for cTBS (*F*_3,60.2_ = 6.450, *p* = 0.001) and not iTBS (*F*_3,60.2_ = 1.494, *p* = 0.225). Planned comparisons revealed significant differences between all categorical measures to one another, except for sleep consolidation and subjective influence and sleep drive and vigilance (*sleep drive* vs. sleep consolidation, *p* = 0.016, vs. psychomotor vigilance, *p* = 0.305, vs. subjective influence, *p* = 0.047; *sleep consolidation* vs. psychomotor vigilance, *p* = 0.005, vs. subjective influence, *p* = 0.712; *psychomotor* *vigilance* vs. subjective influence, *p* = 0.002). Thus suggesting that the pattern of group by location differences described above was indeed specific to sleep consolidation and the resulting subjective influence on the participants.

### Effect on specific measures

**Figure 3:**
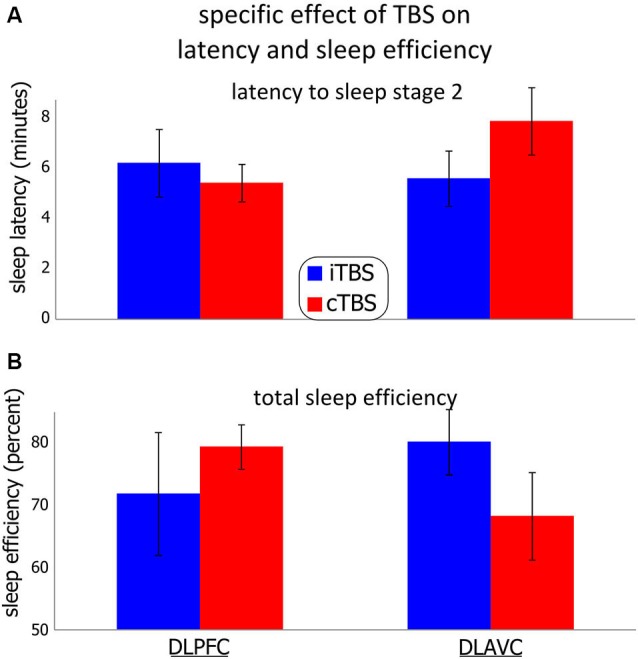
**Effect of theta-burst stimulation on the participants’ latency to non-REM stage 2 and total sleep efficiency**. Two theta-burst protocols were used, continuous (cTBS), and intermittent (iTBS) over the left dorso-lateral prefrontal cortex or the left associative cortex in different sessions. **(A)** (Top) shows the participant’s mean latency to sleep stage 2. **(B)** (Bottom) shows the mean total sleep efficiency, measured as the percentage of total time asleep in any sleep stage over the total time spent in bed. Error bars indicate the standard error for each measurement. See the results section as well as Tables 1 and 2 for statistical details.

Table 2 presents an overview of the mixed model analysis examining each measure of vigilance and the effect of nap time, type of TBS stimulation, the location and their interaction. No measure of vigilance showed a significant main effect of TBS type, but as with the analysis of the categorical measures, several measures indicated significant interactions between the type and location of TBS supporting the results from the categorical measures above. The lack of any significant effect for the three-way interactions including session time for those parameters indicates that the effects of TBS were relatively consistent throughout the day. Both the N2 latency and total sleep efficiency contributed to the significant higher order category “sleep consolidation”, and it is thus unsurprising that each measure also shows significant interaction when examined individually. Further examination of the interaction in N2 latency (Figure 3A) revealed the interaction was primarily driven by differences between dlPFC and the dlAVC for continuous TBS (mean difference = 2.46 min, *SE* = 0.842; *F*_1,40.9_, *p* = 0.006). However, differences in the TBS type stimulation of the dlAVC will have also contributed to the interaction (*F*_1,51.0_, *p* = 0.040).

**Table 2:** **Statistical overview: effects of stimulation for each measured parameter**.

	Descriptive		Mixed model analysis			
Parameter	Mean value	TBS-Type effect	Nap	TBS-Type	Type by location	Nap by type by location
MWT Microsleep	10.99	0.76	*F* = 1.451	*F* = 1.952	*F* = 1.639	*F* = 0.404
	0.816	1.82	*p* = 0.245	*p* = 0.180	*p* = 0.213	*p* = 0.925
MSLT N1 Latency	3.42	2.09	*F* = 2.627	*F* = 0.008	*F* = 1.816	*F* = 1.122
	0.37	1.29	*p* = 0.064*^t^*	*p* = 0.928	*p* = 0.183	*p* = 0.366
MSLT N2 Latency	6.19	3.35	*F* = 2.563	*F* = 0.675	*F* = 4.439	*F* = 1.212
	0.51	1.56	*p* = 0.069*^t^*	*p* = 0.421	*p* = 0.020*	*p* = 0.310
MSLT Sleep Efficiency	75.1	−15.4	*F* = 2.347	*F* = 0.254	*F* = 3.518	*F* = 1.023
	2.8	10.0	*p* = 0.084*^t^*	*p* = 0.619	*p* = 0.041*	*p* = 0.434
PVT Reaction	286.1	−22.3	*F* = 0.583	*F* = 0.254	*F* = 1.350	*F* = 1.081
	11.1	10.7	*p* = 0.630	*p* = 0.620	*p* = 0.268	*p* = 0.399
PVT Fastest 10%	199	−3.76	*F* = 0.524	*F* = 0.101	*F* = 0.450	*F* = 1.745
	3	4.7	*p* = 0.667	*p* = 0.754	*p* = 0.640	*p* = 0.101
PVT Slow 10%	491	−183.9	*F* = 1.261	*F* = 0.700	*F* = 1.681	*F* = 1.823
	33	93.7	*p* = 0.300	*p* = 0.413	*p* = 0.204	*p* = 0.097
Stanford Sleepiness	4.1	−0.39	*F* = 2.494	*F* = 2.103	*F* = 1.485	*F* = 0.408
	0.2	0.43	*p* = 0.073*^t^*	*p* = 0.162	*p* = 0.242	*p* = 0.924
Perceived Latency	7.15	3.35	*F* = 2.076	*F* = 1.528	*F* = 3.459	*F* = 1.816
	0.61	1.7	*p* = 0.117	*p* = 0.231	*p* = 0.046*	*p* = 0.183
Perceived Duration	7.02	−3.10	*F* = 1.386	*F* = 2.051	*F* = 3.770	*F* = 0.890
	0.69	1.49	*p* = 0.260	*p* = 0.168	*p* = 0.034*	*p* = 0.541

The first three columns indicate which parameter was measured, followed by the overall mean value for that parameter, irrespective of stimulation protocol or location, then the maximum measured effect size of each protocol in the same units. Italiced below these values is the measure of the standard error for these values. The last 4 columns indicate the statistical strength and significance level for the main effect of nap, and stimulation type, as well as the 2 and three way interaction examining the effect of location. t indicates a trend level significance while * is indicative of a statistically significant result.

Sleep efficiency showed a similar pattern of results (Figure 3B), in that the effect of cTBS in particular between locations was significantly different (mean difference = 11.01%, *SE* = 5.12%; *F*_1,34.0_ = 4.676, *p* = 0.038), with dlPFC showing more total sleep during the sessions compared to stimulation of the dlAVC. Unlike for the N2 latency, the differences for TBS type was not significant for either location independently, although stimulation of the dlAVC showed the largest TBS type dependent difference (Mean difference 13.01%, *SE* = 6.88%; *F*_1,55.6_ = 3.608, *p* = 0.063). In such a case, the interaction is driven by the reversal of the effects of each TBS type for the dlPFC and dlAVC.

For the subjective measures, the general pattern reflected those of the MSLT measures, indicating that the participant’s subjective experience was simply an accurate reflection of the objective measures of the sleep tests. For both perceived latency and sleep duration, the significant interaction likely reflected that both continuous TBS had differential effects between the locations (mean difference of perceived latency = 2.45 min, *SE* = 0.915; *F*_1,42.9_ = 7.715, *p* = 0.011; mean difference for perceived duration = 2.500 min, *SE* = 0.892; *F*_1,50.2_ = 7.861, *p* = 0.007). To a similar degree, the differences in TBS type over the dlAVC also influenced the interaction (mean difference of perceived latency = 3.18 min, *SE* = 1.29; *F*_1,51.1_ = 6.101, *p* = 0.017; mean difference for perceived duration = 3.51 min, *SE* = 1.42; *F*_1,42.0_ = 6.163, *p* = 0.017).

### Effect of session times

**Figure 4:**
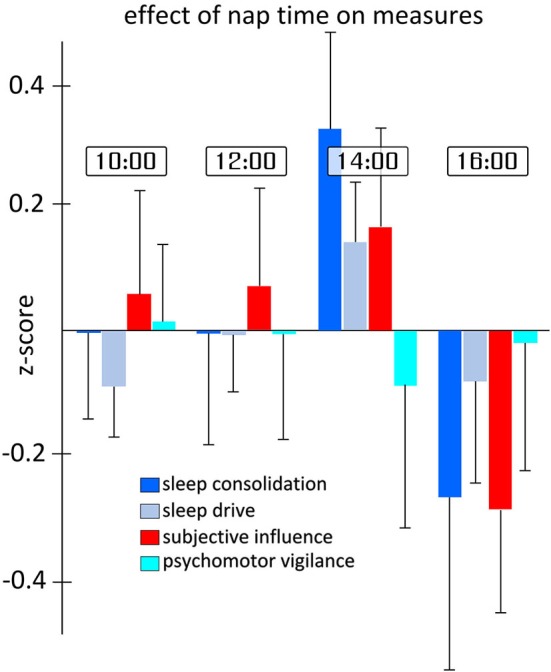
**Differences in sleep measures for each nap time**. Each measure represents the aggregated *z*-scores from various measurements during the day of testing independent of the TBS-type and location of stimulation. As can be seen in the figure, and supported by statistical analysis, the largest differences in measures occur between naps 3 and 4. Error bars indicate the standard error for each measure and nap time independently.

Figure 4 shows the effect of the time of the measurement on each of the four categorical variables. A main effect of nap would indicate that, regardless of any TBS intervention, parameters show differences throughout the experimental day from 10 am to 4 pm. Given that the multivariate analysis showed a significant main effect of nap time (*F*_12,240.1_ = 1.834, *p* = 0.044), we investigated the effect of nap time on each measure individually in order to determine which variable(s) contributed to the effect. Although no main nap effect reached significance, it is noteworthy that the categorical variables “sleep consolidation” and “subjective influence”, all of the individual MSLT measures, as well as the SSS, showed trends towards significance with *p*-values under 0.1. For sleep consolidation (*F*_3,53.1_ = 2.635, *p* = 0.059), *post-hoc* comparisons showed the highest differences in scores between nap 3 and nap 4 (*p* = 0.002). For the effect of nap time on subjective influence (*F*_3,56.1_ = 2.654, *p* = 0.057), pairwise comparisons also found the highest difference between nap 3 and 4 (*p* = 0.034).

The trend found for the three-way interaction for the slowest 10% of PVT reaction times was not explored in depth given the potential complexity of the three-way interaction, as well as the relatively weak statistical support considering none of the other related PVT measures showed similar patterns. Exploratory analysis suggested the trend was most likely related to unusually long reaction times found for the second nap after dlAVC iTBS.

## Discussion

In this exploratory study we found evidence for a dissociable effect of TMS on vigilance for both cTBS and iTBS over two distinct cortical areas. For our categorical measures, both the “sleep consolidation” measure, comprised of both the latency to N2 and the total sleep efficiency as well as the measures for “subjective influence” of stimulation was found to be significantly affected by TBS. Specifically, cTBS over the dlAVC increased latency to N2 and decreased the overall sleep efficiency during the nap compared to cTBS of the dlPFC or iTBS over both locations; and the subjective measures accurately reflected those changes. The effect was particularly pronounced for cTBS compared to iTBS. Contrary to hypothesis, TBS over the occipital cortex, not the frontal cortex, showed the largest effect on our measures for stimulation type and location. Importantly, neither the participant’s drive to sleep, as measures by the initial sleep latencies in the MWT or MSLT, nor their sustained attention in the PVT were affected by either TBS type or the location of stimulation.

### Effect specificity

The finding that TBS only affected certain MSLT measures related to sleep consolidation, but not MWT or PVT, is important for several reasons. Firstly, it is thought that a participant’s performance on the MWT is dependent on personal motivation, the inclined posture during the test, and is subject to various particular strategies to remain awake (Arand et al., 2005), while the MSLT is essentially dependent on a person’s level of sleepiness alone. Thus, the effect of TBS was unlikely to be related to particular motivational aspects, or strategy selections, but instead is likely to have made its impact directly on the participant’s sleepiness. In the same respect, PVT measures also remained unaffected by TBS. Secondly, we did not find a significant effect of TBS on initial sleep latency to N1, but one was found for total sleep efficiency which includes stages of N1. Since cTBS of the dlPFC increased sleep efficiency but participants consistently entered N1 around the same time we assume a stabilizing effect of TBS on sleep. Vice versa, since N1 latencies are consistent, cTBS of the dlAVC destabilizes sleep probably due to increased chances of awakenings after initial sleep onset. The consistent effect of TBS on both, increased sleep propensity and sleep maintenance, point to a common sleep promoting/depressing mechanism induced by TBS. Given that dissociable sleep promoting effects depend on stimulation location, we argue for a sleep promoting cortical to subcortical network that can be influenced by TBS over distinct areas.

Importantly, we found no differences in the effects of TBS based on the time of day, ranging from 10 am to 4 pm. This lack of effect is of interest since both circadian rhythm and increases of homeostatic sleep drive are known to be the basic drivers of both objective and subjective sleepiness (Borbély and Achermann, 1999). Moreover, the consistent trends for overall shorter latencies in the third session are indicative of real changes in the vigilance state of participants throughout the experiment. Increases in homeostatic pressure throughout the day would predict the shortest latencies in the final session; thus leaving circadian factors as the most likely candidate to explain this dip; albeit the finding that MSLT measures during the time range of the experiments have usually been found to be fairly impartial to circadian effects (Dinges et al., 1997).

Results also showed that effects were most robust for comparisons involving cTBS in terms of the differential in location effects and statistical confidence. There are three potential candidate hypotheses to explain the stronger results for cTBS. Firstly, there were more participants receiving cTBS as opposed to iTBS. This may certainly account for the reduced variability in cTBS measures, but not necessarily the clearly larger location differential. Secondly, as Huang et al. (2005) originally reported for the effect of TBS on the motor evoked potential, cTBS is less individually variable than iTBS and its effect remains more stable and consistent over time. In their study on the influence of TBS protocols on the motor evoked potential, iTBS effect peaked 5 min after stimulation and then steadily decreased its effect until becoming insignificant at 25 min, whereas the cTBS protocol reaches its peak effect after 15 min and continues to be significant up to 60 min post-stimulation. Lastly, the largest measured effect of cTBS was to increase the latency of N2 following dlAVC stimulation, that is, to improve the wakefulness of these partially sleep restricted participants; it seems plausible that it is easier to induce wakefulness in sleepy participants than it is to further reduce the latency. Hence potential floor effects may be responsible for larger differences between locations following cTBS.

### Effect strength

Changes in latency to N2 of 3.4 min between type of TMS stimulation and location are similar to results from pharmacological intervention trails. Studies which have used the MWT or MSLT to investigate a various vigilance-altering substances have reported mean latency increases of 2.4 min for modafinil (200–400 mg), and 4.2 min for caffeine (75–400 mg), with a 1.8 min decrease for flurazepam (15–30 mg), (Wesensten et al., 2005). Although it is not the purpose of this study to advocate TBS as a possible clinical treatment for daytime sleepiness or insomnia, it should be noted that the side effects of TMS are relatively low compared to some of the currently accepted treatments (Grossheinrich et al., 2009).

### Effect location

Although there were some differences found between TBS inhibition (cTBS), or excitation (iTBS) of the dlPFC, these were often only significant in their relation to the effects of TBS over the dlAVC; whereas the individual effects found for the dlAVC were the largest in terms of both size and statistical confidence. Thus, contrary to a-priori hypotheses, this region of the occipital cortex was shown to be an inappropriate control site for the stimulation of the frontal regions. Given that we thus lack a true control condition, the pattern of results may be interpreted in two ways. Either the effect of cTBS over the dlAVC produces the largest changes to the natural progression of sleep consolidation, or that iTBS to both locations, along with cTBS to the dlPFC produce the largest and all equivalent effects. Given that previous research strongly supports the differentiation of effects for the two TBS types, including our own differential effects found here for dlAVC stimulation, we support the former conclusion of the two interpretations (Huang et al., 2005; Ko et al., 2008). In the original study using this site as a control (Strafella et al., 2001), no further information was given on the choice of the location except that its stimulation did not result in significant changes to dopamine levels in the caudate nucleus. This finding indicates that the effects found in this study are unlikely to be linked to changes in sub-cortical dopamine levels as research on dlPFC-stimulation would have suggested.

Although previous findings around the dlAVC in relation to sleep are sparse, the connection between this region and sleep deprivation studies in particular are not completely novel. In one of the first imaging studies to examine the effect of sleep deprivation and task ability (serial addition/subtraction task), using positron-emission-topography, Thomas et al. reported significant changes to glucose metabolism in Brodmann areas 39 and 37 described as the inferior parietal to inferior temporal regions (Thomas et al., 2000). In fact, the first PET study describing metabolism before and after sleep deprivation only found significant changes in the posterior temporal and occipital lobes (Wu et al., 1991), but did not describe regions more specifically. Using a logical reasoning task in the fMRI environment, Drummond et al. (2004) found increased activity in those same areas which were a unique response to sleep deprivation. In their study, effect sizes in these regions were larger in the left hemisphere. Finally, this area was shown to be preferentially active after sleep deprivation during a visuo-motor task (Strangman et al., 2005). Again, this fMRI study found that the left hemisphere was particularly sensitive to sleep deprivation. Given the tasks involved were fairly heterogeneous, it seems unlikely that the activity reported was specifically related to task-demands but more characteristic of cortical changes in response to increased levels of sleepiness generally.

### Limitations

This study, being exploratory, has four principle limitations. Firstly, the exploratory nature of the experiment lead to a small sample size overall, but especially when considering the significance of between-subject variable interactions. Given the variability of sleep parameters in general, there is no doubt that a larger cohort of participants will be needed to confirm the findings presented here, and would possibly benefit from a longer within-subject manipulation of TBS-type and more sessions throughout the day. Secondly, given our findings that dlAVC stimulation shows significant differences between the two stimulation types, the study then lacks a sufficient control condition for the results found. In this sense, we lack a baseline value in which to compare our results to. However, given the variability of the participant’s mean latencies, partly due to the high level of individual differences inherent to vigilance measures and also because of the sleep restriction introduced by the experiment, the crucial aspect in this research is not the influence of TBS type and location on baseline values, but the finding that there are significant differences between these conditions. Further studies replicating this effect or exploring different cortical areas would do well to include a sham TBS condition where no direct changes to cortical excitability are to be expected.

Thirdly, in order to ensure that the participants fell asleep at some point during the measures, a certain amount of sleep restriction was necessary. Thus, we cannot necessarily ascribe our results to healthy, non-sleepy participants, nor the perhaps different kind of vigilance changes present in sleep disorders. Nevertheless, the sleep limitations used in this study is fairly mild in comparison to the amount of total deprivation normally seen in studies needed to induce a main significant effect. A more careful monitoring of the participant’s sleep habits prior to the experimental day, even by a relatively simple means such as actigraphy, would have been more helpful in determining the influence of the specific sleep restriction endured by each participant.

Lastly, although cTBS and iTBS have been shown to have inhibitory and excitatory effects respectively over several areas of the cortex (Huang et al., 2005), including the regions of the frontal cortex examined here (Ko et al., 2008), the precise effect of these protocols has not been demonstrated for the dlAVC. In order to determine by which mechanism the TBS protocols over the dlAVC functioned to affect sleep parameters, the subsequent excitability of the underlying cortex as well as the influence on effectively connected structures should be examined in detail. Both of these issues could be resolved using combined TMS-EEG paradigms (Huber et al., 2013). Furthermore, studies have reported an enhancing effect of TBS stimulation if sessions are performed at 10–15 min intervals (Nyffeler et al., 2006, 2009). Although the intervals used in this experiment were much longer (2 h for different locations and 4 h for the same location), repeated stimulation to the same location may have influenced the results found. Further research would benefit from manipulating interval length between repeated TBS sessions.

## Conclusion

Taken together, the specific effects to the latency to N2, along with changes to the overall sleep efficiency in the naps point to a direct effect of the TBS protocols on mechanisms of sleep consolidation which warrant further studies. This is opposed to an effect of the sleep initiation or wake/attention promoting systems as both the latency to N1, the MWT, and the PVT measures were not affected by TBS. In this sense, the null results for those measures in support of earlier studies on the effects of TMS on sleep and vigilance (Cohrs et al., 1998; Graf et al., 2001; Rosenquist et al., 2013). Although this study cannot determine the physiological mechanism of such an effect, it may not be unlike that of sodium oxybate at certain doses. Studies have shown that sodium oxybate act to stabilize sleep and increase the proportion of slow-wave sleep in long-term sleep studies, while at the same time have little to no acute effect on measures of psychomotor vigilance (Ferrara et al., 1999; Oliveto et al., 2010). However, if participants are given the opportunity to sleep for a longer period after taking the drug, there is a significant effect on subsequent latencies to N1 and PVT reaction times (Walsh et al., 2010). Therefore, further studies may wish to examine the effect of TBS on prolonged naps, and whether the effect of sleep consolidation by TBS has a significant effect on post-sleep measures of vigilance.

## Author contributions

Study conception and design: Mensen, Nicklaus, Kuske and Khatami; Acquisition of data: Mensen, Gorban, Nicklaus and Kuske; Analysis and interpretation of data: Mensen, Gorban and Khatami; Drafting of the manuscript: Mensen and Khatami.

## Conflict of interest statement

The authors declare that the research was conducted in the absence of any commercial or financial relationships that could be construed as a potential conflict of interest.
